# Impaired Speckle-Tracking-Derived Left Ventricular Longitudinal Strain Is Associated with Transposition of Great Arteries in Neonates: A Single-Center Study

**DOI:** 10.3390/ijerph20010674

**Published:** 2022-12-30

**Authors:** Daniela Toma, Dorottya Gabor-Miklosi, Andreea Cerghit-Paler, Carmen Corina Șuteu, Marius-Catalin Cosma, Claudiu Mărginean, Mihaela Iancu, Liliana Gozar

**Affiliations:** 1 Emergency Institute of Cardiovascular Diseases and Transplantation, 540139 Târgu-Mureș, Romania; 2 Department of Pediatrics,”George Emil Palade” University of Medicine, Pharmacy, Science, and Technology of Târgu-Mureș, 540139 Târgu-Mureș, Romania; 3 Department of Obstetrics and Gynecology,”George Emil Palade” University of Medicine, Pharmacy, Science, and Technology of Târgu-Mureș, 540139 Târgu-Mureș, Romania; 4 Department of Medical Informatics and Biostatistics, Faculty of Medicine, “Iuliu Hațieganu” University of Medicine and Pharmacy Cluj-Napoca, 400349 Cluj-Napoca, Romania

**Keywords:** transposition of great arteries, speckle tracking, echocardiography, myocardial strain, congenital heart disease, pediatric cardiology

## Abstract

The transposition of great arteries (TGA) is one of the most frequent and severe congenital heart diseases. After newborn stabilization and while pending surgical correction, echocardiographic monitoring with a careful evaluation of left ventricle (LV) performance is warranted. In this study, our objectives were (i) to compare myocardial function, assessed via speckle-tracking echocardiography, between neonates with TGA and neonates without TGA and (ii) to identify a strain parameter with a good discriminatory ability for TGA. We conducted a retrospective, single-center study. A total of 90 neonates were examined, of whom 66 were included (16 comprised the TGA group and 50 comprised the control group). The results of a bivariate analysis showed that classic echocardiography parameters displayed no significant differences between the two studied groups (*p* = 0.785 for EF, *p* = 0.286 for MAPSE and *p* = 0.315 for TAPSE). We found a statistically significant difference between the two groups for the mean values of the LVpGLS parameter (adjusted *p* = 0.0047), with impaired LV myocardium function being observed in the TGA group after adjusting for other covariates. Regarding segmental strain, the mean medial and apical inter-ventricular septum strain values were found to be significantly lower in the neonates with TGA than in the controls (95% CI for difference in means: [−6.45, −0.65], [−8.56, −1.97]). The results of an ROC analysis showed that LVpGLS had a significant ability to differentiate between neonates with TGA and controls (AUC = 0.712, 95% CI: [0.52, 0.903], *p* = 0.011). In conclusion, LVpGLS is a parameter with a significant discriminatory ability for LV dysfunction, and it is useful in the evaluation of ventricular myocardial function in newborns with TGA.

## 1. Introduction

The transposition of great arteries (TGA) is one of the most frequent and severe and, at the same time, one of the most mysterious congenital heart diseases (CHDs). The lesion is defined by ventriculoarterial discordance in the setting of atrioventricular concordance. The most common form of TGA is D-TGA, in which the right ventricle (RV) is positioned to the right of the left ventricle (LV), and the origin of the aorta is situated anterior and to the right of the origin of the pulmonary artery [1,2]. With a prevalence of approximately 2.5/10,000 live births in Europe, it is the fourth most common type of major heart defect [1,2]. According to the associated cardiac anomalies or functional defects that are often seen in patients, TGA is divided into two subtypes: simple TGA and complex TGA. Simple TGA is defined by a group of lesions that includes the following: a patent foramen ovale (PFO), a small persistent ductus arteriosus (PDA) and a small/hemodynamically insignificant ventricular septal defect (VSD). Complex TGA is defined by the association of other major anatomical anomalies, such as a wide/hemodynamically important VSD, a significant subpulmonary stenosis or a coarctation of the aorta (CoAo), e.g., [3,4]. Two parallel circulations result from ventriculoarterial discordance; more precisely, deoxygenated blood from the right atrium is sent back into the systemic circulation, and oxygenated blood from the pulmonary veins is taken back into the pulmonary circulation [5]. In this setting, immediate postnatal therapy is aimed towards ensuring efficient mixing between the two circulations via the maintenance of ductal patency with prostaglandin E1 (PGE1) and atrial septostomy [6,7]. Once the newborn is stabilized, performance of the surgical correction is recommended within the first 3 weeks of life [8]. During this period, alongside the physiological decrease in pulmonary arteriolar resistance, the LV mass also begins to decrease (although with a certain degree of reversibility); thus, echocardiographic monitoring with a careful evaluation of the performance of the LV is warranted [6,9]. A reduction in the indexed LV mass below 35 g/m^2^, the age of the newborn over 3 weeks, A 2D “banana-shaped” echo appearance of the LV, the absence of a PDA or LV outflow tract obstruction represent non-invasive indication criteria for performing an arterial switch operation (ASO) in two stages [10].

Single-stage ASO is currently considered the procedure of choice for newborns with simple TGA. There is a potential risk of myocardial injury after the reimplantation of the coronary arteries, as well as a sudden pressure overload, to which the LV is subjected after the interruption of the cardiopulmonary bypass [7].

We sought to evaluate myocardial function using 2D-strain analyses with the following objectives: (i) to compare longitudinal and segmental strain parameters between two groups, namely, newborns with TGA and newborns without TGA, and (ii) to identify strain parameters with a sensitivity towards the detection of myocardial dysfunction in the group of newborns with TGA (in order to identify a strain parameter with a good discriminatory ability for TGA).

## 2. Materials and Methods

This retrospective study was performed between January 2020 and September 2022 in the Children’s Cardiology Clinic, the Emergency Institute of Cardiovascular Diseases and Heart Transplantation, in Târgu Mureș.

Inclusion criteria: newborns diagnosed with simple TGA and with a complete echocardiographic evaluation performed preoperatively under the conditions of hemodynamic stability (with PDA kept patent via PGE1 infusion, efficient mixing between the two parallel circulations, balloon atrial septostomy performed in the first 24–48 h of life and echocardiographic evaluation performed 48–72 h postnatally and without acidosis). Thus, 20 newborns were included.

Exclusion criteria: newborns diagnosed with complex TGA, with severe hypoxia (O2 saturation below 75%), with metabolic acidosis and/or with inefficient interatrial mixing. Thus, 2 newborns with hemodynamic instability and inefficient mixing due to pulmonary hypertension, which required the administration of nitric oxide, and 2 newborns whose echocardiographic images were of poor quality for strain analyses were excluded.

**Figure 1:**
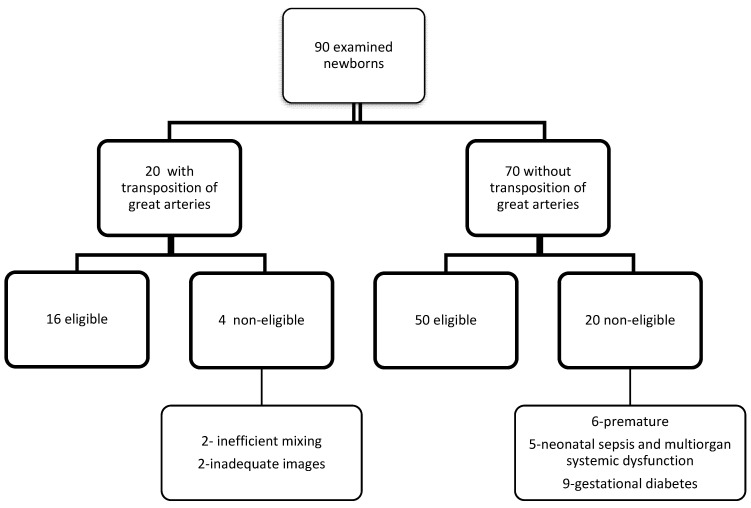
Study flowchart.

Control group (or neonates without TGA) inclusion criteria: full-term, healthy newborns (with no evidence of sepsis, renal impairment or other comorbidities) with no major congenital anomalies, with no history of maternal gestational diabetes and with normal echocardiography (newborns with a PFO or a small atrial septal defect (ASD) were not excluded). Thus, 70 newborns were included in the control group, and premature newborns, as well as those with cardiac and non-cardiac congenital anomalies, were excluded from the study (6 were premature, 5 had neonatal sepsis and multiorgan systemic dysfunction, and 9 had gestational diabetes) (Figure 1).

In order to echocardiographically evaluate the patients, we used an EPIQ 7 ultrasound machine. The monitored echocardiographic parameters included mitral and tricuspid annular plane systolic excursion (MAPSE and TAPSE, respectively), LV EF-Teichholz and the indexed LV mass. Two-dimensional acquisitions from an apical four-chamber view recorded at a frame rate exceeding 70 Hz and optimal quality were stored in the Digital Imaging and Communications in Medicine (DICOM) file format and analyzed offline with Philips QLAB 15 software using LV and RV autostrain functions. In order to analyze an entire cardiac cycle, the M-mode function was used to determine the motion (closing and opening) of the mitral valve, automatically generated using the software. Three points were tagged at the endocardium level (the base of the septum and the lateral and apical locations). The endocardial margin was automatically traced and then manually checked and corrected.

Thus, the pre-procedural images of the 16 included newborns were analyzed by a single investigator, measuring the LV peak longitudinal strain (LVpGLS), RV free-wall longitudinal strain (RVFWSL), RV 4-chamber strain (RV4SLC) and the biventricular segmental strain. The interventricular (InterV) septum and the walls were divided automatically into 3 segments (LV basal, LV medial and LV apical; InterV basal, InterV medial and InterV apical; and RV basal, RV medial and RV apical). The same echocardiographic parameters were examined in the control group.

## 3. Results

### 3.1. Statistical Analysis

Descriptive measures, including arithmetic mean values, sample standard deviations, medians with interquartile ranges (IQRs = [25th percentile, 75th percentile]) and frequencies, are presented for each studied group (TGA group vs. control group). The fitting of the sample data to normal distribution was tested using the Shapiro–Wilk test and by visually inspecting quantile plots (Q-Q plots).

To test for significant differences between the groups (TGA group vs. control group), we compared the mean scores for the overall longitudinal strain variables (LVpGLS, RVFWSL and RV4CSL) of the neonates with TGA with those of the controls by conducting a one-way multivariate analysis of variance (MANOVA). The MANOVA assumption concerning the homogeneity of covariance matrices was tested using Box’s M test, with a significant result being achieved when *p*-value < 0.001. Because there were three response variables, when reporting the MANOVA results, we used Wilks’ lambda test statistic as a more robust test for the unbalanced number of observations in the groups [11].

In order to estimate the group effect size in the MANOVA analysis, we used the partial eta squared ($\;\eta_{p}^{2}$) coefficient, which was interpreted using Cohen’s rules [12] as follows: a value of $\;\eta_{p}^{2}<0$.01 denoted the absence of a group effect, a small effect was obtained if $\;\eta_{p}^{2}\;$ was between 0.01 and 0.06, a medium effect was obtained if $\;\eta_{p}^{2}$ was between 0.06 and 0.14, and a large effect was defined for $\;\eta_{p}^{2}$ > 0.14.

The effect of TGA on longitudinal strain variability was tested in the presence of demographic, anthropometric and clinical covariates using a multiple linear regression analysis in order to determine whether TGA is an independent factor for longitudinal strain variability. Because of the potential collinearity between the independent variables included in the linear regression model, the variance inflation factor (VIF) was estimated. All tested independent variables showed a value of VIF lower than 3.

In order to assess the ability of the longitudinal strains to differentiate between the neonates with TGA and the neonates without TGA, a univariate analysis based on the Receiver Operating Characteristic (ROC) curve was used. The indices for the performances of the longitudinal strains, such as specificity, sensitivity, positive predictive value, negative predictive value, cut-off value and area under the curve (AUC), were derived from ROC analyses using the Youden’s index.

A comparison between regional strain variables (LV strain measurements, InterV strain measurements and RV strain measurements from basal, apical and medial locations) were performed using Student’s t test, assuming equal variances, or the Welch two-sample t-test.

All two-sided statistical tests used a significance level (α) chosen a priori at 0.05, a significant result being defined using an estimated significance level (*p*-value) lower than 0.05.

All statistical analyses were performed in R software version 4.2.2 (R Foundation for Statistical Computing, Vienna, Austria).

### 3.2. Description of Baseline Characteristics of Studied Groups

A sample of 66 subjects, comprising 50 neonates without TGA (controls) and 16 neonates with TGA, was recruited. The neonates’ characteristics are described in Table 1. All neonates without TGA had normal values for LV and RV systolic function. In the group without TGA, 18 (36%) neonates had a PFO, and 32 (64%) had an atrial septal defect.

Both studied groups were comparable regarding all demographic and anthropometric characteristics (*p* > 0.05), although a lower body surface area (BSA) mean was observed (0.21 ± 0.01 vs. 0.22 ± 0.02, *p* = 0.013) in the TGA group.

### 3.3. Group Comparison of Longitudinal Strains (LVpGLS, RVFWSL and RV4CSL)

**Figure 2:**
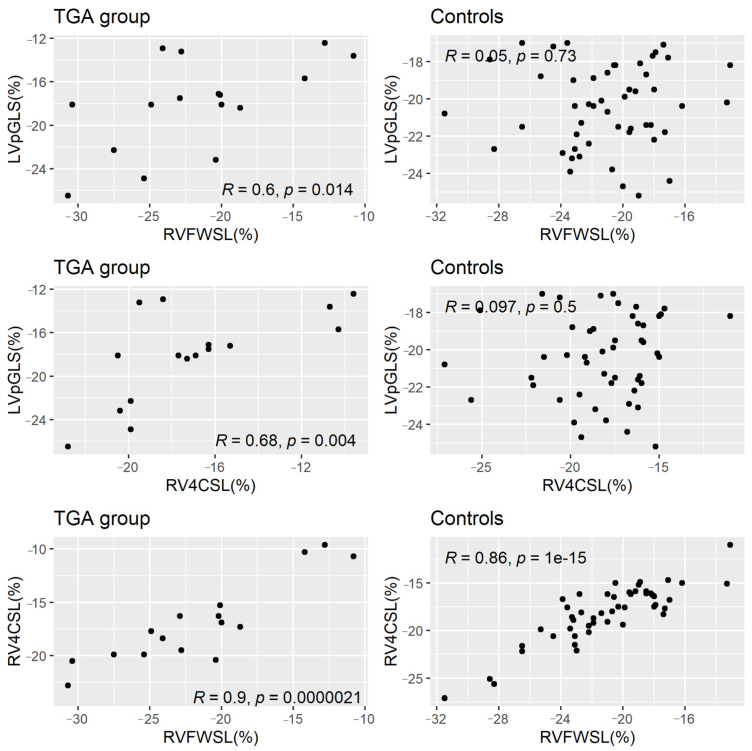
Scatter plots between the left- and right-ventricle-related longitudinal strain variables stratified by neonates with TGA and controls. TGA: transposition of great arteries; LVpGLS: left ventricle peak longitudinal strain; RVFWSL: right ventricle free-wall longitudinal strain; RV4SLC: right ventricle 4-chamber strain.

The linear correlations between LVpGLS, RVFWSL and RV4CSL in all samples are displayed in Table 2. LVpGLS was significantly positively correlated with both RVFWSL (*p* = 0.027) and RV4CSL (*p* = 0.002), while the RV-related longitudinal strain variables had a strong significant linear correlation between them (*p* < 0.0001). After stratification by group, the linear correlations remained statistically significant in the neonates with TGA but not in the controls (Figure 2).

The results of Mardia’s test confirmed the multivariate normality in the total sample (skewness statistics = 11.52, *p* = 0.319, and kurtosis statistics = 1.25, *p* = 0.213) and in each group (*p* > 0.05).

The one-way MANOVA performed on the longitudinal strain variables showed that group had a significant effect (Wilks’ Lambda Λ = 0.77, F(3, 62) = 6.23, *p* = 0.009). The measure of effect size (partial eta squared, $\;\eta_{p}^{2}$) was equal to 0.23, suggesting that group had a large effect on the longitudinal strain variables.

The post hoc one-way ANOVAs carried out following the MANOVA highlighted that the neonates with TGA differed significantly from the controls regarding LVpGLS but not in terms of RVFWSL or RV4CSL (Table 3). Furthermore, the values of LVpGLS were decreased in the neonates with TGA as compared to those in the controls (Table 3).

**Figure 3:**
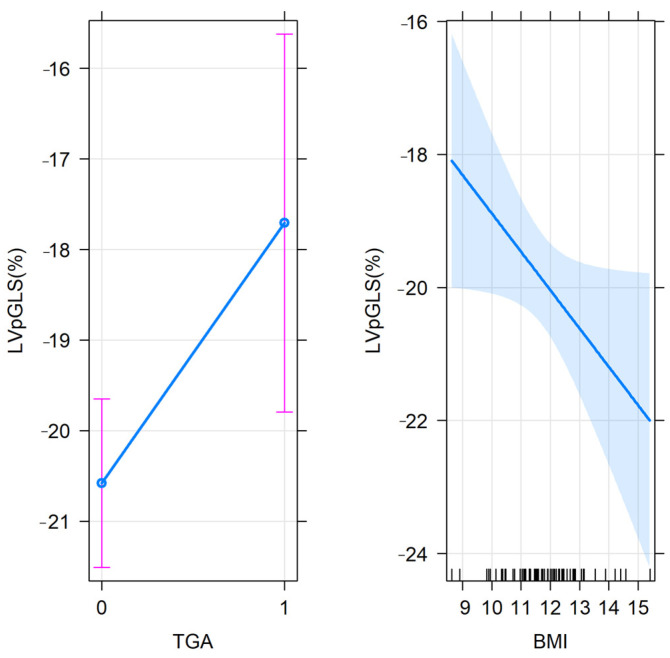
Predictor effect plot for TGA and BMI in the tested multivariable regression model. Note: the graph describes the partial slopes (blue line) for BMI and TGA (groups: 0 = neonates without TGA, and 1 = neonates with TGA), the pointwise confidence band for the regression fitted values (shaded area) and the error bars for the mean values of LVpGLS in the studied groups (neonates with or without TGA). LVpGLS = left ventricle peak longitudinal strain.

After performing the multiple linear regression analysis and adjusting for other covariates, namely, age, BMI, systolic blood pressure, heart rate and indexed left ventricular mass, the association between the left ventricle peak longitudinal strain (LVpGLS) and TGA remained significant despite the significant effect of BMI (Table 4). The magnitude of the adjusted effect of TGA on LVpGLS is described in Figure 3.

### 3.4. Capacity of LVpGLS to Discriminate between TGA and Controls

The results of the ROC analysis showed that LVpGLS had a significant ability to differentiate between neonates with TGA and controls (AUC = 0.712, 95% CI: [0.52, 0.903], *p* = 0.011), with a sensitivity of 75% (95% CI: [48%, 93%]), a specificity of 76% (95% CI: [62%, 87%]), a positive predictive value (PPV) of 50% (95% CI: [34%, 81%]) and a negative predictive value (NPV) of 90% (95% CI: [74%, 95%]) at an optimal cutoff value of −18.40%.

### 3.5. Group Comparisons of Regional Strains (LV Basal, LV Medial, LV Apical, InterV Basal, InterV Medial, InterV Apical, RV Basal, RV Medial and RV Apical)

The regional mean strain values stratified by studied groups are described in Table 5. We found a significant difference between the means of the inter-ventricular septum strain values obtained from the medial (*p* = 0.019) and apical locations (*p* = 0.002). Moreover, the inter-ventricular septum strain mean measured from the medial portion was significantly lower in the neonates with TGA than in the controls (95% IC for difference: [−6.45, −0.65]). We also found that the inter-ventricular septum strain mean measured from the apical location was significantly lower in the neonates with TGA than in the controls (Table 5).

## 4. Discussion

Ventricular function in the neonatal period is difficult to evaluate and interpret due to characteristic changes in the transition from fetal to postnatal circulation, a complex process that involves the closure of fetal shunts and a decrease in pulmonary resistance, resulting in important modifications in both ventricular preload and afterload [13].

The anatomical changes described in neonates with TGA submit the LV to a volume overload in the presence of a decreased afterload, while the RV is subjected to systemic vascular resistance, which results in hypertrophy and the modification of the ventricular shape, with an increase in its sphericity and the displacement of the interventricular septum towards the LV [8]. LV afterload decreases in the immediate postnatal period along with the decrease in pulmonary resistance, which could result in a decline in LV performance. Some echocardiographic aspects, such as the InterV septum bulging towards the LV, resulting in a “banana-shaped” appearance, and an LV mass below 35 g/m², are suggestive of an LV incapable of supporting systemic circulation [14]. However, Foran et al., in their study conducted in newborns with TGA who were operated after the time frame considered to be optimal, claim that they were unable to identify a predictor for unfavorable postoperative evolution and that changes in the geometry of the LV, secondary to the decrease in pulmonary resistance, do not indicate modifications of the LV myocardium [15].

In conclusion, although an accurate evaluation of the ventricular myocardial dysfunction in newborns with this pathology represents an extremely important factor, especially in borderline situations when it is primordial for optimal decision making regarding surgical management, until now, no other echocardiographic parameters had been identified that could accurately evaluate ventricular performance.

Moreover, Walter et al. published a study wherein they extensively analyzed classic echocardiographic parameters in fetuses and newborns with TGA compared to those in healthy fetuses and newborns, respectively. They found a statistically significant difference between certain biventricular morphological and functional parameters in fetuses with TGA and those of their healthy counterparts. After analyzing these parameters independently, the fetuses and newborns with TGA showed normal biventricular function that was superior when compared to that of healthy fetuses and newborns [16].

In our study, several parameters obtained via classic echocardiography were analyzed. All the parameters submitted for analyses, more specifically, EF in the M mode, MAPSE and TAPSE, were within the normal limits for age and weight, and no statistical significance was found between the two groups. Nevertheless, an evaluation of systolic function through the M mode does not express uniform LV systolic function and, thus, gives little information regarding global function [17]. In addition, the value of the indexed LV mass obtained for the group of newborns with TGA was statistically significantly higher than the value obtained for the control group. Given the fact that the echocardiography was performed, without exception, in the first 72 h after birth, it is obvious that the decrease in ventricular mass in the presence of this pathology does not occur early. Likewise, the initial increase in the ventricular mass in the group of newborns with TGA is explained by the hemodynamics of the heart malformation, which involves the adaptation of the two ventricles to both pressure and volume overload under the conditions of early increased pulmonary vascular resistance postnatally. Therefore, this compensatory mechanism masks progressive left ventricular dysfunction, making it impossible to identify and quantify using classic echocardiographic parameters. It is only known, and has been previously proven, that a reduction in the indexed LV mass of 35 g/m² and a banana-shaped appearance are predictors of unfavorable evolution, associated with increased mortality and postoperative morbidity [18], but these changes appear after the age of 3 weeks.

Regarding the results obtained via the speckle-tracking analysis, we found a statistically significant difference between the two groups for the LVpGLS parameter, a difference that can attest to a decrease in the performance of the LV myocardium in the group of newborns with TGA. When comparing the segmental strain values obtained from the apical four-chamber view, statistically significant differences were obtained at the level of the InterV septum and the apical and median segments. A difference with a tendency towards statistical significance was also recorded at the level of the basal segment of the LV lateral wall. No statistically significant differences were obtained regarding the segments of the RV lateral wall.

Although the classic echocardiography parameters did not differentiate in terms of left ventricular function between the two groups and the indexed LV mass values were even higher in the TGA group, some of the longitudinal strain parameters were effective in revealing the impaired ventricular function in the TGA group.

The LVpGLS value proved to be statistically significantly lower in the newborns with TGA than in those in the control group, independent of the BMI value, the EF value and the LV mass, which strengthens the hypothesis that this strain parameter (LVpGLS) can be used to predict the clinical evolution of TGA [19,20].

## 5. Conclusions

Our study provides evidence that a speckle-tracking analysis is useful for evaluating the ventricular myocardial function in newborns with TGA, representing a promising approach for evaluations of cardiac function. LVpGLS differentiated those with TGA from those without TGA, but conclusions concerning the magnitude of its diagnostic ability require further studies in larger samples. The use of LVpGLS as a predictive parameter for LV dysfunction could be applied in the perioperative management of patients with TGA.

## 6. Limitations

Our study has several limitations: (i) The data analysis was based on a relatively small number of patients with TGA in a retrospective, single-center study, which might have limited the power to detect subtle cardiac dysfunction; therefore, a prospective, multicenter, multivendor study is required to validate our results for clinical application. (ii) The diagnostic ability of LVpGLS was based on a univariate analysis; therefore, a multivariate analysis taking into consideration other conventional cardiac imaging markers is required to test its independent discriminatory capacity.

## Figures and Tables

**Table 1:** Baseline characteristics of neonates without and with TGA.

Variables	Neonates without TGA (n_1_ = 50)	Neonates with TGA (n_2_ = 16)	*p*-Value
Age (days) ^a^	3 [2, 4.75]	2 [2, 3]	0.210
Gender (male) ^c^	32 (64)	12 (75)	0.417
Heart rate (bpm) ^b^	132.1 ± 10.5	136.7 ± 11.1	0.139
Birth weight (kg) ^b^	3.42 ± 0.40	3.33 ± 0.39	0.482
BMI (kg/m^2^) ^b^	11.51 ± 1.02	12.43 ± 1.80	0.067
BSA (m^2^) ^b^	0.22 ± 0.02	0.21 ± 0.01	0.013 *
Apgar score at 1 min ^a^	9 [9, 10]	8 [8, 8]	<0.0001 *
Apgar score at 5 min ^a^	10 [10, 10]	9 [8, 9]	<0.0001 *
SaO2 (%) ^a^	99 [99, 100]	85 [84.75, 86.50]	<0.00001 *
Systolic blood pressure (mmHg) ^a^	72.5 [65.5, 76.0]	84 [78.75, 92]	<0.0001 *
Diastolic blood pressure (mmHg) ^a^	36 [35, 45]	45 [40, 48.5]	0.014 *
Gestational age (weeks) ^a^	38.5 [38, 39]	39 [39, 39]	0.169
C-section ^c^	9 (18)	9 (56.2)	0.003 *
Pathological pregnancy ^c^	NA	7 (43.75)	NA
Genetic syndrome ^c^	NA	0 (0)	NA
Conventional echocardiographic parameters			
EF ^b^	69.42 ± 6.54	69.06 ± 3.68	0.785
MAPSE (mm) ^a^	6.7 [6.2, 7.9]	6.9 [6.7, 7.0]	0.286
TAPSE (mm) ^a^	9 [7.8, 9.7]	8 [7.7, 9.0]	0.315
Indexed left ventricular mass (g/m^2^) ^a^	57.39 ± 4.68	68.15 ± 12.32	0.003 *

Data are presented as ^a^ median [IQR], IQR = interquartile range defined by lower (Q1) and upper quartiles (Q3); ^b^ arithmetic mean ± standard deviation or ^c^ number of cases (n) and relative frequencies (%). NA = not available. * statistical significance: *p*-value < 0.05; *p*-values were obtained from Welch two-sample t-tests, t-test assuming equal variances or Mann–Whitney. SaO2 (%) = oxygen saturation in the arterial blood; Apgar score at 1 min = Apgar score at 1 min after birth; Apgar score at 5 min = Apgar score at 5 min after birth; C-section = birth by cesarean section; PS requiring prostaglandin infusion = patient with severe pulmonary stenosis requiring treatment soon after birth with prostaglandin infusion; EF = ejection fraction M mode; MAPSE = mitral annular plane systolic excursion; TAPSE = tricuspid annular plane systolic excursion.

**Table 2:** Pearson correlation coefficient matrix between the longitudinal strain values for all samples of neonates (*n* = 66).

	LVpGLS	RVFWSL	RV4CSL
LVpGLS	1	0.27 [0.03, 0.48]	0.37 [0.14, 0.56]
RVFWSL		1	0.85 [0.77, 0.90]
RV4CSL			1

Sample estimates represent correlation coefficient (r) and 95% confidence interval (CI) for r. LVpGLS = left ventricle peak longitudinal strain; RVFWSL = right ventricle free-wall longitudinal strain; RV4CSL = right ventricle 4-chamber strain.

**Table 3:** One-way analysis of variance (ANOVA) with group (neonates with TGA vs. neonates without TGA) as between-subject factor performed on the left- and right-ventricle-related longitudinal strain variables.

	Neonates without TGA (n_1_ = 50)	Neonates with TGA (n_2_ = 16)	Group (with TGA vs. without TGA)		
	M (SD)	M (SD)	F(1, 64)	*p*	$\;\eta_{p}^{2}$
LVpGLS (%)	−20.46 (2.22)	−18.08 (4.26)	8.60	0.0047 *	0.12
RVFWSL (%)	−18.11 (2.97)	−16.99 (3.88)	1.49	0.2264	0.02
RV4CSL (%)	−21.03 (3.63)	−21.62 (5.75)	0.24	0.6269	0.004

Note: M = sample arithmetic mean; SD = sample standard deviation; F = Fisher’s statistics; *p* = estimated significance level (*p*-value); * statistically significant results (*p* < 0.05); $\;\eta_{p}^{2}$ = partial eta squared; TGA = transposition of great arteries; LVpGLS = left ventricle peak longitudinal strain; RVFWSL = right ventricle free-wall longitudinal strain; RV4CSL = right ventricle 4-chamber strain.

**Table 4:** Multivariable linear regression model for longitudinal strain measurements (LVpGLS) on overall sample (*n* = 66).

Independent Variables	Unstandardized $\hat{\beta }$ Partial Regression Coef-Ficients	ES	Standardized $\hat{\beta }$ Partial Regression Coef-Ficients	*p*-Value	VIF
TGA group	2.87	1.30	2.21	0.031 *	2.65
Age (years)	0.34	0.27	0.15	0.257	1.21
BMI (kg/m^2^)	−0.58	0.29	−0.25	0.049 *	1.16
Systolic blood pressure (mmHg)	0.04	0.06	0.10	0.559	2.26
Heart rate (bpm)	0.03	0.03	0.11	0.413	1.44
Indexed left V = ventricular mass (g/m^2^)	−0.03	0.05	−0.10	0.506	1.57
Adjusted R^2^ = 0.14, F(6,59) = 2.75, *p*-value = 0.02					

ES = standard error of estimated regression coefficients; VIF = variance inflation factor; TGA = transposition of great arteries. * statistically significant results (*p* < 0.05).

**Table 5:** Distributions of regional strain measurements in studied groups (neonates with TGA vs. neonates without TGA).

	Neonates without TGA (n_1_ = 50)	Neonates with TGA (n_2_ = 16)	Groups (with TGA vs. without TGA)	
	M (SD)	M (SD)	*p*-Value	95% IC for Difference in Means
LV strain measurements (%)				
Basal	−30.35 (8.67)	−26.22 (7.54)	0.093	[−8.96, 0.70]
Medial	−12.83 (4.68)	−12.66 (4.57)	0.878	[−2.87, 2.46]
Apical	−19.35 (7.14)	−19.02 (6.82)	0.871	[−4.39, 3.72]
InterV strain measurements (%)				
Basal	−15.64 (6.47)	−13.28 (4.46)	0.176	[−5.86, 1.10]
Medial	−19.53 (3.39)	−15.98 (5.20)	0.019 *	[−6.45, −0.65]
Apical	−27.45 (5.51)	−22.18 (6.45)	0.002 *	[−8.56, −1.97]
RV strain measurements (%)				
Basal	−22.95 (4.30)	−21.98 (6.06)	0.482	[−3.70, 1.77]
Medial	−19.74 (4.19)	−20.83 (5.30)	0.400	[−1.48, 3.65]
Apical	−19.30 (4.08)	−19.59 (6.18)	0.859	[−3.15, 3.75]

Note: M = sample arithmetic mean; SD = sample standard deviation; *p* = estimated significance level (*p*-value) obtained from Student’s t test, assuming equal variances, or Welch two-sample *t*-test; * statistically significant results (*p* < 0.05); TGA = transposition of great arteries; LV = left ventricle; InterV = interventricular septum.

## Data Availability

The raw data presented in this study can be obtained upon reasonable request to Liliana Gozar at lili_gozar@yahoo.com.

## References

[1] David A.K. L-Transposition of the Great Arteries: Anatomy, Physiology, Clinical Features, and Diagnosis. Last Updated: 19 September 2022. https://www.uptodate.com.

[2] Giang K.W., Mandalenakis Z., Fedchenko M., Eriksson P., Rosengren A., Norman M., Hanseus K., Dellborg M. (2022). Congenital heart disease: Changes in recorded birth prevalence and cardiac interventions over the past half-century in Sweden. Eur. J. Prev. Cardiol..

[3] Shaddy R.E., Penny D.J., Feltes T.F., Cetta F., Mital S. (2022). Transposition of the Great Arteries. Moss and Adams’ Heart Disease in Infants, Children, and Adolescents: Including the Fetus and Young Adult.

[4] Park M.-K., Salamat M. (2021). Cyanotic Congenital Heart Defects. Park’s Pediatric Cardiology for Practitioners.

[5] O’Byrne M.L., Glatz A.C., Song L., Griffis H.M., Millenson M.E., Gillespie M.J., Dori Y., De Witt A.G., Mascio C.E., Rome J.J. (2018). Association between variation in preoperative care before arterial switch operation and outcomes in patients with transposition of the great arteries. Circulation.

[6] Sarris G.E., Balmer C., Bonou P., Comas J.V., da Cruz E., Di Chiara L., Di Donato R.M., Fragata J., Jokinen T.E., Kirvassilis G. (2017). Clinical guidelines for the management of patients with transposition of the great arteries with intact ventricular septum. Cardiol. Young.

[7] Crepaz R., Secchieri S., Svaluto G., Milanesi O., Pitscheider W., Gentili L., Rubino M., Stellin G. (1997). Echocardiographic evaluation of systolic and diastolic left ventricular function following arterial switch operation in the neonatal period for transposition of the great arteries. Midterm results. G. Ital. Cardiol..

[8] Séguéla P.E., Roubertie F., Kreitmann B., Mauriat P., Tafer N., Jalal Z., Thambo J.B. (2017). Transposition of the great arteries: Rationale for tailored preoperative management. Arch. Cardiovasc. Dis..

[9] Abuelkasem E., Wang D.W., Omer M.A., Abdelmoneim S.S., Howard-Quijano K., Rakesh H., Subramaniam K. (2019). Perioperative clinical utility of myocardial deformation imaging: A narrative review. Br. J. Anaesth..

[10] Busro P.W., Sani A.A., Caesario M. (2020). A successful management of late-presenting transposition with intact septum and severe coarctation of the aorta. SAGE Open Med. Case Rep..

[11] Ateş C., Kaymaz Ö., Kale H.E., Tekindal M.A. (2019). Comparison of Test Statistics of Nonnormal and Unbalanced Samples for Multivariate Analysis of Variance in terms of Type-I Error Rates. Comput. Math Methods Med..

[12] Cohen J. (1988). Statistical Power Analysis for the Behavioral Sciences.

[13] Schubert U., Muller M., Norman M., Abdul-Khaliq A. (2013). Transition from fetal to neonatal life: Changes in cardiac function assessed by speckle-tracking echocardiography. Early Hum. Dev..

[14] Lacour-Gayet F., Piot D., Zoghbi J., Serraf A., Gruber P., Macé L., Touchot A., Planché C. (2001). Surgical management and indication of left ventricular retraining in arterial switch for transposition of the great arteries with intact ventricular septum. Eur. J. Cardiothorac Surg..

[15] Foran J.P., Sullivan I.D., Elliott M.J., de Level M.R. (1998). Primary arterial switch operation for transposition of the great arteries with intact ventricular septum in infants older than 21 days. J. Am. Coll. Cardiol..

[16] Walter C., Soveral I., Bartrons J., Escobar M.C., Carretero J.M., Quirado L., Gómez O., Sánchez-de-Toledo J. (2020). Comprehensive Functional Echocardiographic Assessment of Transpostion of the Great Arteries: From Fetus to Newborn. Pediatr. Cardiol..

[17] Simma B., Fritz M.G., Trawoger R., Geiger R., Fink C., Hammerer I. (1997). Changes in left ventricular function in shocked newborns. Intensive Care Med..

[18] Goo H.W., PhD M., Park S.H. (2020). Pattern Analysis of Left Ventricular Remodeling Using Cardiac Computed Tomography in Children with Congenital Heart Disease: Preliminary Results. Korean J. Radiol..

[19] Forsey J., Friedberg M., Mertens L. (2013). Speckle Tracking Echocardiography in Pediatric and Congenital Heart Disease. Echocardiography.

[20] D’Andrea A., Cante L., Palermi S., Carbone A., Ilardi F., Sabatella F., Crescibene F., Di Maio M., Giallauria F., Messalli G. (2022). COVID-19 Myocarditis: Prognostic Role of Bedside Speckle-Tracking Echocardiography and Association with Total Scar Burden. Int. J. Environ. Res. Public Health.

